# Incremental value of physiological indices to predict high-risk plaques detected by NIRS-IVUS

**DOI:** 10.1007/s12928-025-01116-7

**Published:** 2025-03-12

**Authors:** Kazuyoshi Kakehi, Masafumi Ueno, Nobuhiro Yamada, Kyohei Onishi, Keishiro Sugimoto, Yohei Funauchi, Takayuki Kawamura, Kosuke Fujita, Hiroki Matsuzoe, Koichiro Matsumura, Gaku Nakazawa

**Affiliations:** Division of Cardiology, Department of Internal Medicine, Kindai University Faculty of Medicine, Ohno-Higashi, Osakasayama, Osaka 589-8511 Japan

**Keywords:** Acute coronary syndrome, Coronary computed tomographic angiography, High-risk plaque, Maximum lipid core burden index, Quantitative flow reserve

## Abstract

**Abstract:**

Identification of vulnerable plaques is important for reducing future cardiovascular events. This study aimed to investigate optimal modalities other than intravascular imaging in evaluating vulnerable plaques. We prospectively evaluated 105 non-culprit coronary lesions by CCTA imaging and near-infrared spectroscopy-intravascular ultrasound in 32 patients with acute coronary syndrome. Angiographically-derived ΔQFR and ΔFFR_CT_ were measured as the difference in QFR and FFR_CT_ across the stenosis. A receiver operating characteristic curve analysis was performed to determine the optimal cutoff values of angiographically- and CCTA-derived plaque features for a maxLCBI_4mm_ ≥ 400. The best cutoff values for ΔQFR and ΔFFR_CT_ to predict a maxLCBI_4mm_ ≥ 400 were 0.05 and 0.06, respectively. ΔQFR and ΔFFR_CT_ values and percent diameter stenosis on QCA or CCTA were associated with a maxLCBI_4mm_ ≥ 400 (both P < 0.05). The combination of ΔFFR_CT_ ≥ 0.06 and plaque density predicted a maxLCBI_4mm_ ≥ 400 with 89.4% sensitivity and 84.5% specificity (area under the curve, 0.90; P < 0.0001). There was no significant difference in area under the curve values between ΔQFR and plaque density + ΔFFR_CT_ ≥ 0.06 (0.92 vs. 0.90, P = 0.50). In the diagnosis of vulnerable plaques in acute coronary syndrome, the combination of ΔFFR_CT_ and plaque density shows a diagnostic capability similar to that of ΔQFR in non-culprit lesions.

**Graphical Abstract:**

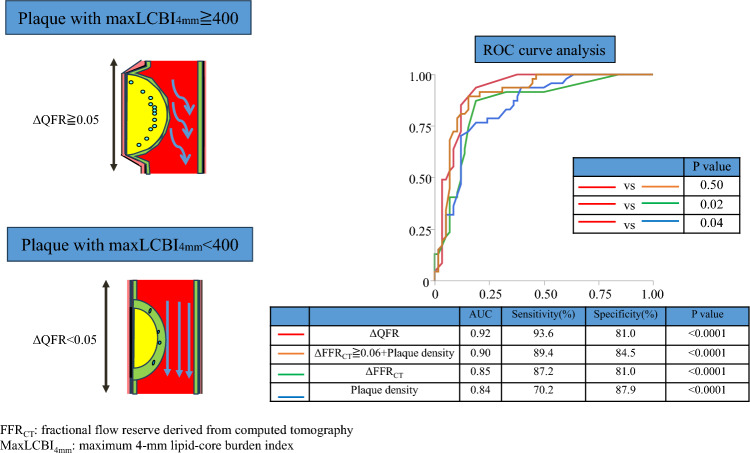

**Supplementary Information:**

The online version contains supplementary material available at 10.1007/s12928-025-01116-7.

## Introduction

Coronary artery disease is a major cause of death globally. Primary percutaneous coronary intervention (PCI) improves the prognosis of patients with acute myocardial infarction, and secondary prevention medications also improve outcomes. However, patients with a history of myocardial infarction remain at a high risk of future cardiovascular events [1]. The incidence of future cardiovascular events after PCI in acute coronary syndrome (ACS) is more frequent with non-culprit lesions than with culprit leisons [2]. In particular, non-culprit lesions in ACS are more likely to have early unplanned revascularization than non-culprit lesions in chronic coronary syndrome [3]. Multivessel disease affects approximately 50% of patients with ACS and is burdened by poor outcomes and high mortality [4]. Myocardial revascularization strategies regarding non-culprit lesions have been extensively discussed in the literature [5].

Most cases of ACS are not necessarily caused by severe stenosis, and in approximately 70% of cases, the degree of coronary artery stenosis before the onset of ACS is < 50% [6]. Autopsy studies have shown that many coronary artery events are due to plaque rupture, and thin-cap fibroatheroma is considered to be a precursor lesion of plaque rupture [7]. Various imaging modalities have been used to detect these precursor lesions. Detecting vulnerable plaques of non-culprit lesions in patients with ACS may help reduce future cardiovascular events. Near-infrared spectroscopy and intravascular ultrasound (NIRS-IVUS) is used to identify vulnerable plaques. The non-culprit lesions of a maximum 4-mm lipid core burden index (maxLCBI_4mm_) ≥ 400, as indicated by NIRS-IVUS, corresponds to cardiovascular events [8], and plaques with a maxLCBI_4mm_ ≥ 400 are considered vulnerable plaques.

Coronary computed tomographic angiography (CCTA) can non-invasively evaluate many of these plaques. In a prospective study with an average follow-up of 27 months, a high incidence of ACS was reported in patients with coronary artery plaques with low-attenuation plaques (mean: < 30 Hounsfield units [HU]) or positive remodeling (remodeling factor > 1.1 as measured by the vessel diameter) on CCTA images [9]. The comprehensive evaluation of plaque vulnerability factors on CCTA may be useful for future risk stratification. Several studies have also reported the use of CCTA results in aggressive secondary prevention [10]. High-risk plaques that cause ACS have been found by noninvasive hemodynamic assessment to have fluid dynamic abnormalities, such as wall shear stress (WSS) and high axial plaque stress [11]. The invasive fractional flow reserve ratio (FFR) is a functional test to guide revascularization with PCI that requires guidewire insertion into coronary arteries and administration of a drug that induces hyperemia. The quantitative flow ratio (QFR) is a novel, accurate method that can estimate the FFR from coronary angiography without wire insertion or drug administration for hyperemia [12]. Fractional flow reserve derived from computed tomography (FFR_CT_) is a non-invasive imaging post-processing technique that uses artificial intelligence to analyze data obtained from conventional CCTA. In the comparison of FFR_CT_ and QFR for lesions with an FFR ≤ 0.8 in intermediate stenosis according to chronic coronary syndrome, QFR showed a better area under the curve (AUC) than FFR_CT_ (0.93 vs 0.82) [13]. In functional evaluation, QFR is considered superior to FFR_CT_. However, there have been no studies on which method is better for identifying vulnerable plaques and how CCTA parameters or FFR_CT_ can be optimized to approach the accuracy of QFR. Therefore, this study aimed to evaluate whether invasive assessment using QFR or non-invasive assessment using FFR_CT_ is more predictive of lipid-rich plaques with a maxLCBI_4mm_ ≥ 400. We also aimed to evaluate the relationship between angiographically-derived plaque features and CCTA-derived plaque features.

## Methods

### Study population and design

In this prospective, observational study, we compared a maxLCBI_4mm_ and CCTA-derived plaque features/FFR_CT_/QFR in non-culprit lesions with ACS. Patients were recruited in the Division of Cardiology at Kindai University Hospital and were enrolled if they met all of the following inclusion criteria: (1) patients were admitted with ACS (ST-segment elevation myocardial infarction, non-ST-segment elevation myocardial infarction, and unstable angina pectoris); (2) intermediate stenosis in non-culprit lesions on coronary angiography or CCTA; (3) NIRS-IVUS evaluation for intermediate stenosis after percutaneous coronary intervention; and (4) each non-culprit lesion was evaluated using CCTA and FFR_CT_ performed within a duration of 14 days between computed tomography (CT) and percutaneous coronary intervention. Patients were excluded if any of the following criteria were encountered: (1) chronic kidney disease as shown by an estimated glomerular filtration rate < 30 ml/min/1.73 m^2^; (2) hemodynamic instability; (3) severe valvular heart diseases, (4) left main trunk lesions; (5) lesions that could not be evaluated by NIRS-IVUS for any reason (e.g., tortuous vessels, severe stenosis, and calcification that made catheter passage difficult); and (6) poor image quality that resulted in an inability to perform CCTA or NIRS-IVUS analysis. Each coronary artery was divided into 30-mm long segments beginning at the ostium to evaluate the vulnerability of lesions [14]. A non-culprit lesion was defined as intermediate stenosis with no history of percutaneous coronary intervention and no 30-mm segment of the responsible lesion. Lesions with 25%–70% stenosis on quantitative coronary angiography (QCA) and CCTA were defined as intermediate stenosis. Coronary angiography was performed using a radial or femoral artery approach using a 6 Fr sheath and catheter. Three major vessels, namely the left anterior descending artery, left circumflex artery, and right coronary artery, were examined by NIRS-IVUS, after treating the culprit lesion in ACS.

### NIRS imaging

**Fig. 1:**
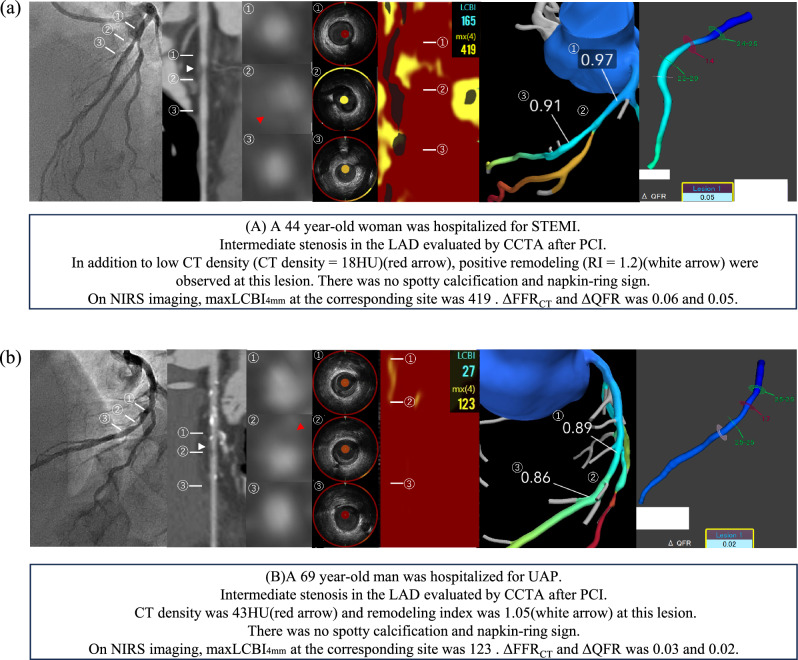
Representative cases. *CCTA* coronary computed tomography, *CT* computed tomography, *FFR*_*CT*_ fractional flow reserve derived from computed tomography, *HU* Hounsfield units, *LAD* left anterior descending, *maxLCBI*_*4mm*_ maximum 4-mm lipid-core burden index, *NIRS* near-infrared spectroscopy, *PCI* percutaneous coronary intervention, *QFR* quantitative flow ratio, *RI* remodeling index, *STEMI* ST-elevation myocardial infarction, *UAP* unstable angina pectoris

The NIRS system used in this study consisted of a 3.2 F rapid exchange catheter, pullback and rotation device, and a console (Dual pro™; Infraredx, Bedford, MA, USA). Image acquisition was automatically pulled back from the most distal site of the target artery at a rate of 1.0 mm/s and 1800 rpm (Dual pro). Areas of the artery with spectral characteristics of a lipid core were displayed in yellow within the image map (chemogram). The Makoto® system (Infraredx) was used to analyze the obtained chemogram data [15]. Each coronary artery was divided into 30-mm segments beginning at the ostium, and the maxLCBI_4mm_ within the 30-mm segments was measured. The presence of vulnerable plaques was defined as a maxLCBI_4mm_ ≥ 400 [8]. Two representative cases of plaques with a maxLCBI_4mm_ ≥ 400 and < 400 are shown in Fig. 1.

### CCTA imaging

Standard methods of cardiac CT imaging and enhancement were used in this study. An electrocardiogram-synchronized, 512-slice CT system (GE Healthcare, Chicago, IL, USA) was used to perform cardiac CT scans with the test bolus tracking method. Sublingual nitrates were administered before scanning in all patients. If necessary, oral and/or intravenous beta-blockers were administered to maintain the heart rate at < 60 beats/min. A 10-ml test bolus of the contrast agent iopamidol (Iopamiron 370; Bracco Diagnostics, Milan, Italy) was administered to estimate the scan timing. The main bolus of contrast agent (0.8 ml/kg of iopamidol 370) was then injected. The scan was initiated when the ascending aorta was maximally enhanced. The scan parameters were as follows: 120 kV, 500–700 mA, 0.16 helical pitch, 350-ms gantry rotation time, and a slice thickness per interval of 0.625 × 0.625 mm. Cardiac CT images were constructed at 75% of the RR interval.

Coronary plaques were more likely to be vulnerable plaques if they had the following characteristics on CCTA: (1) the presence of non-calcified or low-attenuation plaques with a measured value < 30 Hounsfield units (HU), (2) positive remodeling, (3) spotty calcification, and (4) napkin-ring sign. Spotty calcification was classified as < 3 mm in size on curved multiplanar remodeling images and unilateral on cross-sectional images [16]. Napkin-ring sign was defined as ring-like peripheral higher attenuation of the non-calcified portion of a coronary plaque.

SYNAPSE VINCENT® (Fujifilm Medical Co., Tokyo, Japan) was used to measure the plaque density, remodeling index, and calcification [16]. Plaque density (HU) of the analyzed lesion was calculated by placing several regions of interest (approximately 0.5–1.0 mm^2^) at the plaque site, measuring the plaque density of each region of interest, and averaging them.

Coronary artery remodeling was assessed by calculating the difference in the vessel diameter at the plaque site compared with a reference site in a normal-appearing segment proximal to the lesion, with positive remodeling defined as an index ≥ 1.1.

### FFRCT

Evaluation of plaque vulnerability on CCTA images was performed at Kindai University Hospital, and evaluation of FFR_CT_ was performed using HeartFlow (Redwood City, CA, USA) in the same manner as in the usual insurance practice. FFR_CT_ was calculated from CCTA data using computational fluid dynamics modeling followed by semi-automatic segmentation of the coronary artery and left ventricular mass. Coronary blood flow and pressure were simulated under conditions modeling maximal hyperemia. The details of the principles underlying the FFR_CT_ calculations have been reported previously [17].

Delta (Δ) FFR_CT_ represents the change in FFR_CT_ across a stenosis and was measured as the difference between FFR_CT_ proximal and distal to the stenosis (i.e., ΔFFR_CT_ = proximal FFR_CT_ − distal FFR_CT_).

### QFR

Three-dimensional (3D) QCA analysis and QFR computation were performed in a blinded fashion using validated software (QAngio XA 3D version 1.0.28.4; Medis Medical Imaging Systems, Leiden, the Netherlands). Two angiographic projections ≥ 25° apart, which presented the least foreshortening of the stenosis and minimum overlap of the main vessel and side branches, were used for the analysis. In these projections, two end-diastolic frames were selected with electrocardiographic guidance. The investigator identified one or two anatomical landmarks (e.g., bifurcations) as reference points for matching location information in the two frames and subsequently indicated the most proximal site and the most distal site of the vessel. Vessel contours were automatically detected and manually corrected if required. The software reconstructed a 3D anatomical vessel model without its side branches for the 3D QCA and QFR computation. The 3D QCA analysis included a minimum luminal diameter, reference vessel diameter, percent diameter stenosis, and lesion length.

The QFR computation was performed on the basis of anatomical information from 3D QCA using a specific flow model of contrast-flow QFR. Details of the computational method and underlying principle of contrast-flow QFR were previously reported [18]. In short, contrast-flow QFR was computed using a modeled hyperemic flow velocity, based on Thrombolysis In Myocardial Infarction (TIMI) frame count analysis without drug-induced hyperemia. The TIMI frame count analysis was performed on either of the two angiographic projections that provided more well-defined contrast flow.

ΔQFR represents the change in QFR across a stenosis and was measured as the difference between QFR proximal and distal to the stenosis (i.e., ΔQFR = proximal QFR − distal QFR).

### Statistical analysis

The Kolmogorov–Smirnov test was used to determine whether continuous variables had a normal distribution. Continuous variables are presented as the mean ± standard deviation if they had a normal distribution or as the median and interquartile range if the distribution was not normal. Student’s t-test or the Mann–Whitney U test was used for comparisons of continuous variables where appropriate. Categorical variables are expressed as frequencies and percentages. The relationships between maxLCBI_4mm_ and angiographically- and CCTA-derived plaque features were assessed by the Pearson correlation coefficient. Categorical variables were tested by the chi-square test or Fisher’s exact test. A receiver operating characteristic (ROC) curve analysis was performed to determine the optimal cutoff values for angiographically- and CCTA-derived plaque features such as percent diameter stenosis and plaque density for predicting a maxLCBI_4mm_ ≥ 400. A multivariate logistic regression analysis was performed to investigate predictive factors for a maxLCBI_4mm_ ≥ 400. The odds ratio and 95% confidence interval were calculated. All univariate variables with P < 0.05 and those deemed of clinical interest were included in the statistical model. All P values < 0.05 were considered statistically significant. The statistical analysis was performed using JMP Pro software (SAS Institute Inc., Cary, NC, USA).

## Results

### Study population

We enrolled 117 consecutive patients with intermediate stenosis in non-culprit lesions as shown by coronary angiography or CCTA who were admitted for ACS to Kindai University Hospital between December 2021 and January 2024. Of these, 85 patients were excluded for the following reasons: 6 patients failed to have lesions as shown by NIRS-IVUS; 63 patients had coronary angiography but no CCTA; 1 patient had more than a 14-day interval between CCTA and percutaneous coronary intervention; and 15 patients could not have FFR_CT_ performed. Finally, we included 32 patients in the study (Supplemental Fig. 1). Table 1 shows the patients’ characteristics. The patients had a mean age of 68 ± 11.5 years, 84.4% were men, and 53.1% had unstable angina and a high prevalence of risk factors (hypertension: 78.1%, dyslipidemia: 71.9%, and type 2 diabetes mellitus: 40.6%). The mean low-density lipoprotein cholesterol concentration at admission was 117.6 ± 33.6 mg/dl.

**Table 1:** Baseline clinical characteristics

Basic characteristics (n = 32patients)	
Age	68.0 ± 11.5
Male gender, n (%)	27 (84.4%)
Body mass index, (kg/m^2^)	23.1 (21.3–24.8)
Hypertension, n (%)	25 (78.1%)
Diabetes mellitus, n (%)	13 (40.6%)
Dyslipidemia, n (%)	23 (71.9%)
Smoking (past, current), n (%)	22 (68.8%)
*Clinical presentation*	
STEMI, n (%)	12 (37.5%)
NSTEMI, n (%)	3 (9.4%)
UAP, n (%)	17 (53.1%)
*Medication at admission*	
β-blocker, n (%)	7 (21.9%)
ACE-I/ARB, n (%)	11 (34.4%)
Statin, n (%)	4 (12.5%)
Ezetimibe, n (%)	0 (0%)
*Laboratory data*	
eGFR(ml/min/1.73m^2^)	70.8 ± 19.3
HbA1c, (%, IQR)	6.1(6.0–6.8)
Triglyceride, (mg/dl, IQR)	105 (64.5–118)
LDL-cholesterol (mg/dl)	117.6 ± 33.6
HDL-cholesterol (mg/dl)	48.7 ± 10.1
non HDL-cholesterol (mg/dl)	138.8 ± 35.0

Continuous variables are expressed as the mean ± standard deviation

*ACE-I* angiotensin-converting enzyme inhibitor, *ARB* angiotensin II receptor blocker, *eGFR* estimated glomerular filtration rate, *HDL* high-density lipoprotein, *IQR* interquartile range, *NSTEMI* non-ST-elevation myocardial infarction, *STEMI* ST-elevation myocardial infarction, *UAP* unstable angina pectoris

### Angiographically-, NIRS-IVUS-, and CCTA-derived features of the analyzed lesions

Table 2 shows the angiographic characteristics of the analyzed lesions. A total of 43% of analyzed lesions were located within the left anterior descending artery and approximately 50% were proximal lesions.

**Table 2:** Angiographically-, NIRS-IVUS-, and CCTA-derived plaque features

Location of lesions(n = 105lesions)	
LAD artery, n (%)	45 (42.9%)
LCX artery, n (%)	27 (25.7%)
RCA artery, n (%)	33 (31.4%)
Proximal lesion, n (%)	52 (49.5%)
Mid lesion, n (%)	38 (36.2%)
Distal lesion, n (%)	13 (12.4%)
Far distal lesion, n (%)	2 (1.9%)
*Angiographic findings*, *NIRS-IVUS findings*	
% diameter stenosis on QCA, (%, IQR)	45 (33.5–53)
MaxLCBI_4mm_, (IQR)	350 (221–484)
MaxLCBI_4mm_ ≥ 400	46 (43.8%)
*CCTA findings at maxLCBI*_*4mm*_* lumen site*	
Duration of days between CT and PCI(days, IQR)	4.0 (0–8.8)
Vessel area(mm^2^, IQR)	13.7 (9.9–16.7)
Lumen area(mm^2^, IQR)	4.9 (3.3–6.6)
Plaque area(mm^2^, IQR)	8.3 (5.9–10.8)
Plaque density, (HU, IQR)	41 (26.0–60.0)
% diameter stenosis, (%, IQR)	41 (30–51)
Remodeling index, (IQR)	1.0 (0.95–1.1)
Spotty calcification, n (%)	28 (26.7%)
Napkin-ring sign, n (%)	9 (8.6%)
*Physiological measurements at the site of maxLCBI*_*4mm*_	
ΔFFR_CT_, (IQR)	0.05 (0.03–0.12)
ΔQFR, (IQR)	0.04 (0.02–0.14)

*CCTA* coronary computed tomography, *CT* computed tomography, *FFR*_*CT*_ fractional flow reserve derived from computed tomography, *IQR* interquartile range, *LAD* left anterior descending, *LCX* left circumflex, *LDL* low-density lipoprotein, *maxLCBI*_*4mm*_ maximum 4-mm lipid-core burden index, *NIRS-IVUS* near-infrared spectroscopy and intravascular ultrasound, *PCI* percutaneous coronary intervention, *QFR* quantitative flow reserve, *RCA* right coronary artery

In the angiographic and NIRS-IVUS analysis, the percent diameter stenosis on QCA was 45% (33.5–53%), the median maxLCBI_4mm_ was 350 (221–484), and the prevalence of a maxLCBI_4mm_ ≥ 400 was 43.8%. In the CCTA analysis, the median CT value of plaques with maxLCBI_4mm_ in the 30-mm segment was 41 (26–60) HU. In a physiological analysis, the median ΔFFR_CT_ and ΔQFR values were 0.05 (0.03–0.12) and 0.04 (0.02–0.14), respectively.

### Relationships between the maxLCBI_4mm_ and angiographically- and CCTA-derived plaque features

**Fig. 2:**
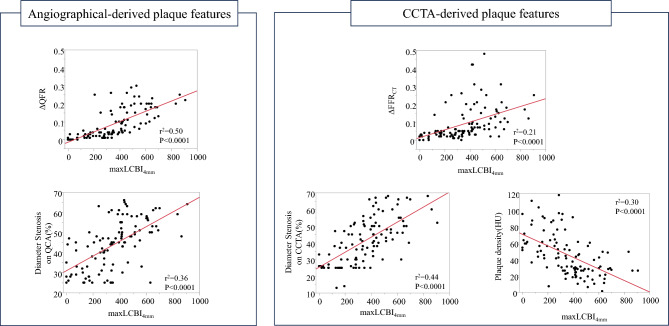
Relationships between the maxLCBI_4mm_ and angiographically- and CCTA-derived plaque features. ΔQFR and ΔFFR_CT_ values, and the percent diameter stenosis on QCA and CCTA were moderately and positively correlated with the maxLCBI_4mm_. Furthermore, plaque density on CCTA was negatively correlated with the maxLCBI_4mm_. *CCTA* coronary computed tomography, *FFR*_*CT*_ fractional flow reserve derived from computed tomography, *HU* Hounsfield units, *maxLCBI*_*4mm*_ maximum 4-mm lipid-core burden index, *QCA* quantitative coronary angiography, *QFR* quantitative flow reserve

The relationships of the maxLCBI_4mm_ at target lesions with angiographically- and CCTA-derived plaque features are shown by scatterplots (Fig. 2). ΔQFR and ΔFFR_CT_ values and the percent diameter stenosis on QCA and CCTA were moderately and positively correlated with the maxLCBI_4mm_ (r^2^ = 0.50, P < 0.001; r^2^ = 0.21, P < 0.001; r^2^ = 0.36, P < 0.001; r^2^ = 0.44, P < 0.001, respectively). Furthermore, plaque density on CCTA was negatively correlated with the maxLCBI_4mm_ (r^2^ = 0.30, P < 0.001). The correlations of ΔQFR and ΔFFR_CT_ with the maxLCBI_4mm_ for each vessel and location were examined. ΔQFR values were more strongly positively correlated with the maxLCBI_4mm_ than ΔFFR_CT_ values for lesions other than distal lesions (Supplemental Fig. 2).

**Fig. 3:**
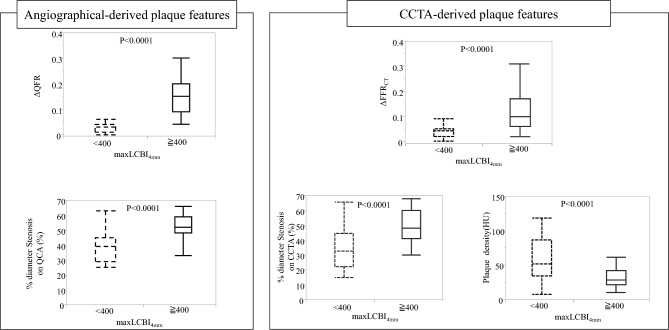
Comparison of angiographically- and CCTA-derived plaque features between analyzed coronary lesions with and without a maxLCBI_4mm_ ≥ 400. Lesions that showed a maxLCBI_4mm_ ≥ 400 were more likely to show larger ΔFFR_CT_ and ΔQFR values and percent diameter stenosis on QCA and CCTA, and a lower plaque density on CCTA than those that showed a maxLCBI_4mm_ < 400. *CCTA* coronary computed tomography, *FFR*_*CT*_ fractional flow reserve derived from computed tomography, *HU* Hounsfield units, *maxLCBI*_*4mm*_ maximum 4-mm lipid-core burden index, *QCA* quantitative coronary angiography, *QFR* quantitative flow reserve

Figure 3 shows the relationships between the maxLCBI_4mm_ and angiographically- and CCTA-derived plaque features. Lesions that showed a maxLCBI_4mm_ ≥ 400 were more likely to have a larger ΔQFR value (0.15 [0.09–0.20] vs. 0.03 [0.01–0.04], P < 0.001) ΔFFR_CT_ value (0.1 [0.06–0.18] vs. 0.04 [0.02–0.05], P < 0.001), and percent diameter stenosis on QCA and CCTA (52 [48–59] vs. 39 [29–45], P < 0.001; 50 [42–60] vs. 31 [25–45], P < 0.0001, respectively), and a lower plaque density (28 [20–33] HU vs. 54 [41–76] HU, P < 0.001) than those that showed a maxLCBI_4mm_ < 400.

**Fig. 4:**
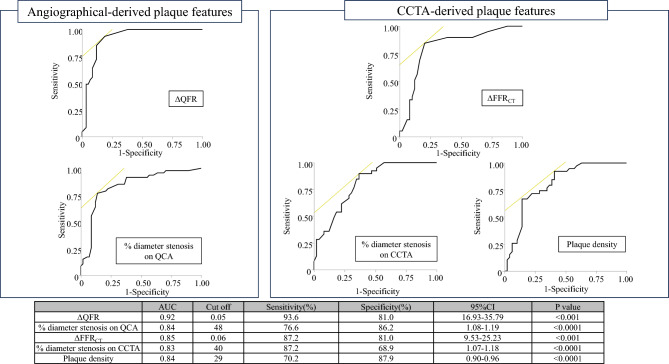
ROC curve analysis for predicting a maxLCBI_4mm_ ≥ 400. An ROC curve analysis was performed to predict a maxLCBI_4mm_ ≥ 400 for ΔQFR, percent diameter stenosis on QCA, ΔFFR_CT_, percent diameter stenosis on CCTA, and plaque density. *AUC* area under the curve, *CCTA* coronary computed tomography, *CI* confidence interval, *FFR*_*CT*_ fractional flow reserve derived from computed tomography, *maxLCBI*_*4mm*_ maximum 4-mm lipid-core burden index, *QCA* quantitative coronary angiography, *QFR* quantitative flow reserve, *ROC* receiver operating characteristic

**Fig. 5:**
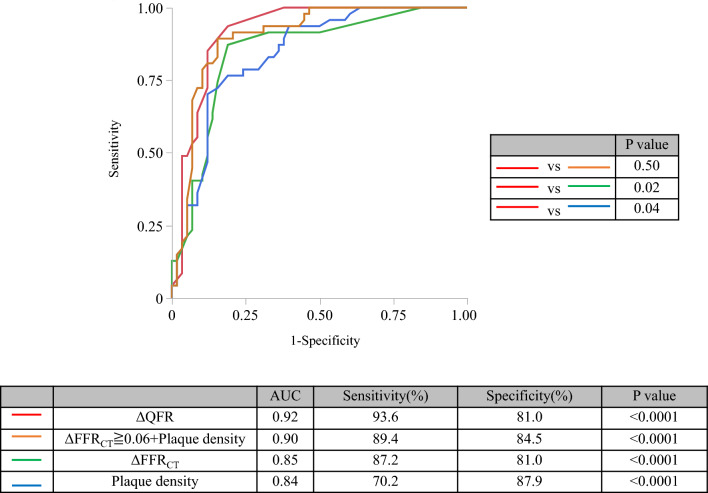
ROC curve analysis based on the combination of ΔFFR_CT_ ≥ 0.06 and plaque density for predicting a maxLCBI_4mm_ ≥ 400. There was a significant difference in AUC values between ΔQFR and ΔFFR_CT_, and between ΔQFR and plaque density. However, there was no significant difference in AUC values between ΔQFR and the combination of plaque density + ΔFFR_CT_ ≥ 0.06. *AUC* area under the curve, *CCTA* coronary computed tomography, *FFR*_*CT*_ fractional flow reserve derived from computed tomography, *maxLCBI*_*4mm*_ maximum 4-mm lipid-core burden index, *QFR* quantitative flow reserve

Figure 4 shows the results of the ROC analysis for predicting a maxLCBI_4mm_ ≥ 400. The ROC analysis showed that ΔQFR ≥ 0.05 (AUC: 0.92, sensitivity: 93.6%, specificity: 81.0%), ΔFFR_CT_ ≥ 0.06 (AUC: 0.85, sensitivity: 87.2%, specificity: 81.0%), percent diameter stenosis on QCA ≥ 48% (AUC: 0.84, sensitivity: 76.6.4%, specificity: 86.2%), percent diameter stenosis on CCTA ≥ 40% (AUC: 0.83, sensitivity: 87.2%, specificity: 69.0%), and plaque density ≤ 29HU (AUC: 0.84, sensitivity: 70.2%, specificity: 87.9%) were optimal cutoff values associated with a maxLCBI_4mm_ ≥ 400. The model of adding a cutoff value of ΔFFR_CT_ ≥ 0.06 to plaque density (plaque density + ΔFFR_CT_ ≥ 0.06) showed the highest discriminative ability of a maxLCBI_4mm_ ≥ 400 (AUC: 0.90, sensitivity: 89.4%, specificity: 84.5%) (Fig. 5). There were significant differences in AUC values between ΔQFR and ΔFFR_CT_ (0.92 vs. 0.85, P = 0.02), and between ΔQFR and plaque density (0.92 vs. 0.84, P = 0.04). However, there was no significant difference in AUC values between ΔQFR and plaque density + ΔFFR_CT_ ≥ 0.06 (0.92 vs. 0.90, P = 0.50). The results of the univariate and multivariate analyses for determining the predictive factors for a maxLCBI_4mm_ ≥ 400 are shown in Table 3. The univariate analysis showed that plaque density, percent diameter stenosis on QCA and CCTA, ΔQFR ≥ 0.05, and ΔFFR_CT_ ≥ 0.06 predicted a maxLCBI_4mm_ ≥ 400 in coronary lesions. In the multivariate analysis of angiographically-derived plaque features, ΔQFR ≥ 0.05 and percent diameter stenosis on QCA were independent predictors of a maxLCBI_4mm_ ≥ 400 (P < 0.001 and P = 0.01, respectively). In the multivariate analysis of CCTA-derived plaque features, ΔFFR_CT_ ≥ 0.06, plaque density, and percent diameter stenosis on CCTA were independent predictors of a maxLCBI_4mm_ ≥ 400 (P = 0.003, P = 0.01, and P = 0.008, respectively).

**Table 3:** Univariate and multivariate logistic analyses of factors for predicting a maxLCBI_4mm_ ≥ 400

	Univariate analysis		Multivariate analysis		Multivariate analysis	
	OR(95%CI)	P value	OR(95%CI)	P value	OR(95%CI)	P value
ΔQFR ≥ 0.05	62.67 (16.39–239.61)	< 0.0001	34.02 (8.43–137.14)	< 0.0001		
% diameter stenosis on QCA (%)	1.14 (1.08–1.19)	< 0.0001	1.08 (1.01–1.14)	0.01		
ΔFFR_CT_ ≥ 0.06	29.20 (9.92–85.92)	< 0.0001			6.97 (1.94–25.02)	0.003
% diameter stenosis on CCTA(%)	1.12 (1.07–1.18)	< 0.0001			1.07 (1.02–1.13)	0.01
Plaque density	0.93 (0.90–0.96)	< 0.0001			0.95 (0.92–0.99)	0.008

*CI* confidence interval, *FFR*_*CT*_ fractional flow reserve derived from computed tomography, *OR* odds ratio, *RI* remodeling index, *QFR* quantitative flow reserve

## Discussion

To the best of our knowledge, this is the first study to show that ΔQFR values are better than ΔFFR_CT_ values for predicting vulnerable plaques with intermediate stenosis of non-culprit lesions in ACS. The main finding in our study is that non-invasive assessment by the combination of ΔFFR_CT_ and plaque density was similar to invasive assessment of ΔQFR for plaque vulnerability.

### Relationships between vulnerable plaques and physiological indices

Previous studies have reported that plaque vulnerability is associated with reduced FFR [19]. Generally, lipid plaques are formed by an influx of atherogenic inflammatory cytokines and oxidative stress, leading to endothelial dysfunction [20]. This mechanism is supported by studies that showed more severe endothelial dysfunction in target lesions containing large amounts of necrotic core material [19]. The endothelium regulates vascular tone [21], and lipid atheroma with endothelial dysfunction may cause an inadequate vascular response during hyperemic conditions, which may lead to an increased ΔFFR value. Previous studies have reported that wall shear stress, axial plaque stress, and the pressure gradient are correlated with ΔFFR_CT_, and there is a strong correlation between shear stress and the site of plaque rupture [22]. Therefore, vulnerable plaques may affect ΔFFR_CT_ or ΔQFR more than stable plaques because of stronger wall shear stress and axial plaque stress, and a higher pressure gradient. Studies using IVUS have suggested that the amount of lipid plaques is related to the FFR value [23]. Additionally, several reports have demonstrated a correlation between the lipid quantity assessed by optical coherence tomography (OCT) and the severity evaluated by FFR [24]. Additionally, there is a correlation between the morphological features of plaques, such as lipid arc and lipid length, assessed by OCT and the severity of FFR [25]. Thin-cap fibroatheroma in non-culprit lesions is correlated with a maxLCBI_4mm_ ≥ 400 and lower QFR [26]. QFR values show good discrimination for the presence of an OCT-minimal lumen area (MLA) < 3.5 mm^2^, IVUS-MLA < 4 mm [2], and a plaque burden ≥ 70% [26]. Compared with the FFR, the QFR can provide incremental efficacy in predicting the presence of Thin-cap fibroatheroma [27]. FFR_CT_ values have been shown to correlate with IVUS-MLA < 4 mm^2^ and a plaque burden ≥ 70% [28]. Previous studies that compared FFR_CT_ with invasive FFR have shown that adding FFR_CT_ to CCTA parameters improves the specificity, positive predictive value, and diagnostic accuracy [29]. In a previous report, the optimal QFR cutoff value for predicting an FFR ≤ 0.80 was 0.8 (AUC: 0.93, sensitivity: 90%, specificity: 82%), and the optimal FFR_CT_ cutoff value for predicting an FFR ≤ 0.80 was 0.79 (AUC:0.82, sensitivity: 81%, specificity 74%). Therefore, QFR is closely correlated with FFR [13]. In this study, in which the percent diameter stenosis on QCA was 45% (33.5%–53%) and the lesion was not severely stenosed, both ΔFFR_CT_ and ΔQFR values were strongly correlated with the maxLCBI_4mm_, but ΔQFR was significantly better in predicting a maxLCBI_4mm_ ≥ 400. Therefore, ΔQFR and ΔFFR_CT_ values may not only be related to the severity of local stenosis in the target lesion, but also may be related to the characteristics of plaques. In this study of intermediate stenosis on CCTA, although noninvasive parameters by CCTA were not better predictors of a maxLCBI_4mm_ ≥ 400 than ΔQFR values, the combination of FFR_CT_ values and plaque density improved the accuracy in predicting a maxLCBI_4mm_ ≥ 400, which was not significantly different to ΔQFR values.

In conclusion, ΔQFR values are strongly related to the presence of vulnerable plaques. However, the combination of ΔFFR_CT_ values and plaques may also have a similar diagnostic ability to that of ΔQFR values.

## Study limitations

This study has some limitations. First, this was a single-center study, and the results should be considered hypothesis-generating only. Second, we did not include patients with ACS who did not consent to the study and some patients in whom CCTA could not be performed or FFRCT could not be measured. Third, as for the cutoff value of the factor predicting maxLCBI_4mm_ ≥ 400 indicating a high possibility of vulnerable plaques, this is only in the acute phase of ACS patients, we have not examined whether similar results can be shown in the chronic phase of ACS patients.

## Conclusion

In the diagnosis of vulnerable plaques in acute coronary syndrome, the combination of ΔFFRCT and plaque density shows a diagnostic capability similar to that of ΔQFR in non-culprit lesions.

## Supplementary Information

Below is the link to the electronic supplementary material.

Supplementary file1 (PPTX 89 KB)

## Data Availability

The authors confirm that the data supporting the findings of this study are available within the article and its supplementary materials.
