# Supplementary material for: Incremental value of physiological indices to predict high-risk plaques detected by NIRS-IVUS
Source: Cardiovasc Interv Ther. 2025 Mar 12;40(3):588–98. doi: 10.1007/s12928-025-01116-7 (PMC12167347; doi:10.1007/s12928-025-01116-7)
Supplement: Supplementary file 1 — Supplementary file1 (PPTX 89 KB) [file 12928_2025_1116_MOESM1_ESM.pptx]

## Slide 1
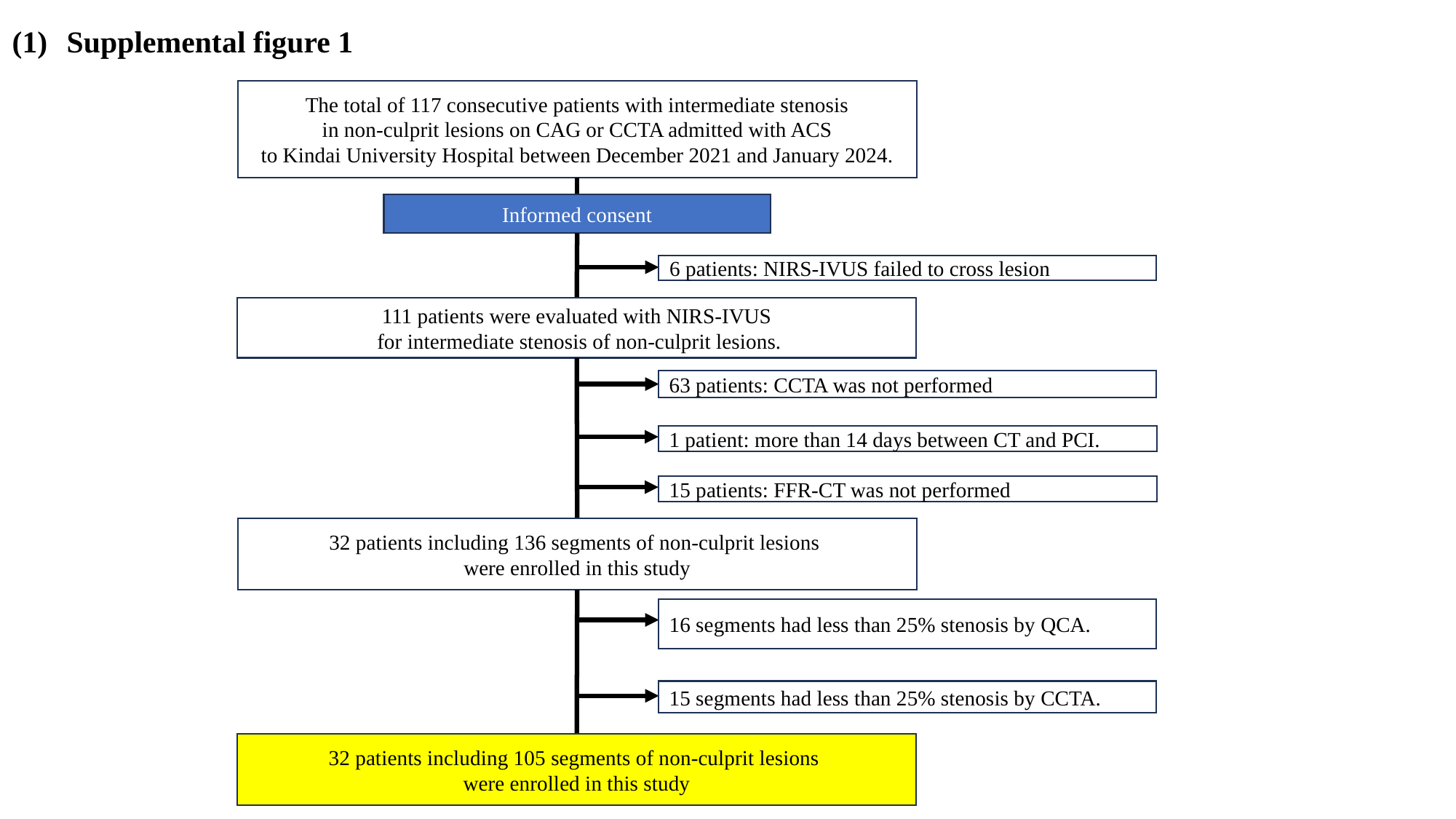

Supplemental figure 1
The total of 117 consecutive patients with intermediate stenosis
 in non-culprit lesions on CAG or CCTA admitted with ACS
to Kindai University Hospital between December 2021 and January 2024.
Informed consent
6 patients: NIRS-IVUS failed to cross lesion
111 patients were evaluated with NIRS-IVUS
 for intermediate stenosis of non-culprit lesions.
63 patients: CCTA was not performed
1 patient: more than 14 days between CT and PCI.
15 patients: FFR-CT was not performed
32 patients including 136 segments of non-culprit lesions
were enrolled in this study
16 segments had less than 25% stenosis by QCA.
15 segments had less than 25% stenosis by CCTA.
32 patients including 105 segments of non-culprit lesions
were enrolled in this study

## Slide 2
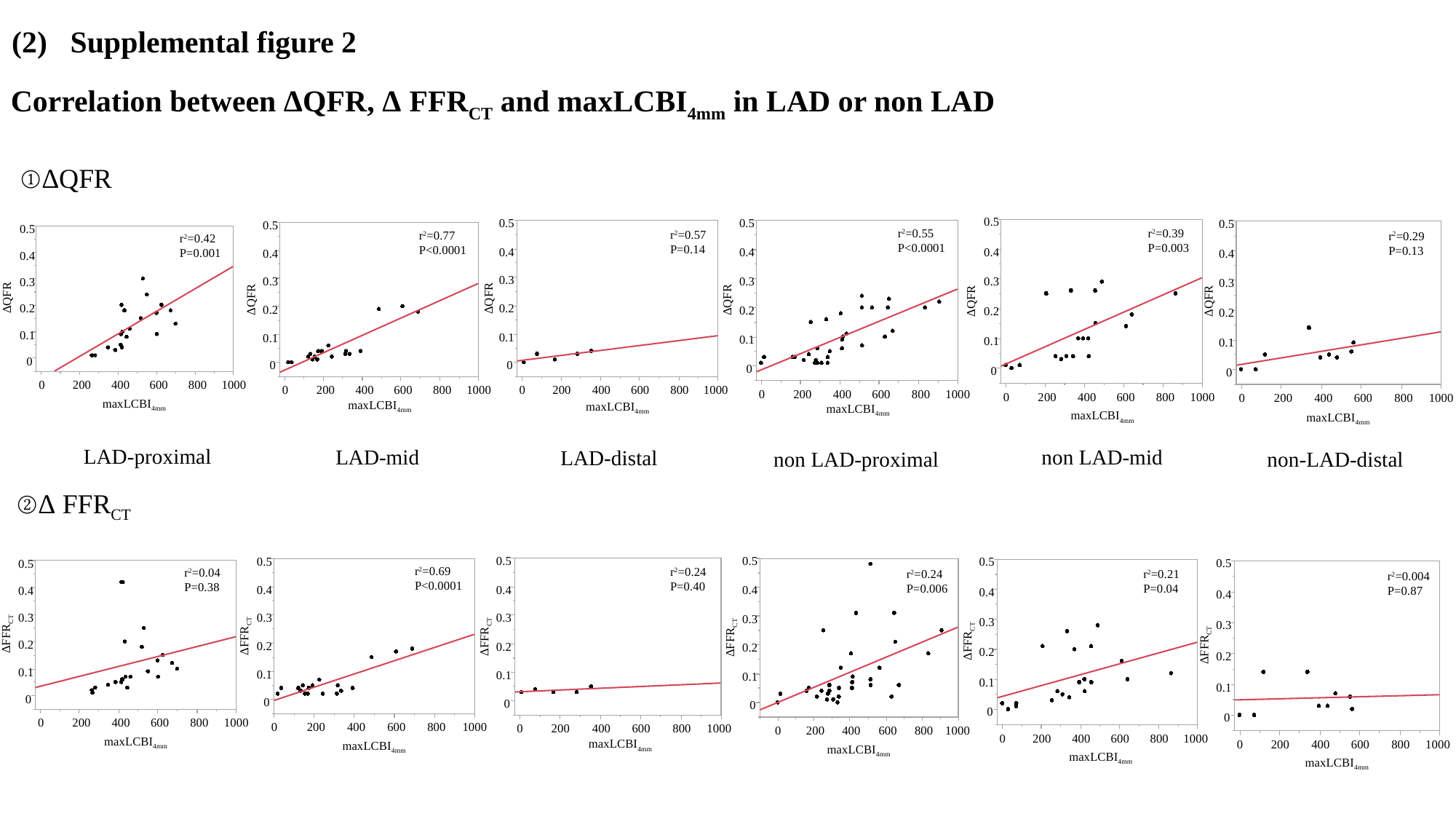

(2) Supplemental figure 2
Correlation between ΔQFR, Δ FFRCT and maxLCBI4mm in LAD or non LAD
①ΔQFR
0.5
0.4
0.3
0.2
0.1
0
0
200
400
600
800
1000
maxLCBI4mm
ΔQFR
0.5
0.4
0.3
0.2
0.1
0
0
200
400
600
800
1000
maxLCBI4mm
ΔQFR
0.5
0.4
0.3
0.2
0.1
0
0
200
400
600
800
1000
ΔQFR
maxLCBI4mm
0.5
0.4
0.3
0.2
0.1
0
0
200
400
600
800
1000
ΔQFR
maxLCBI4mm
0.5
0.4
0.3
0.2
0.1
0
0
200
400
600
800
1000
ΔQFR
maxLCBI4mm
0.5
0.4
0.3
0.2
0.1
0
0
200
400
600
800
1000
ΔQFR
maxLCBI4mm
r2=0.55
P<0.0001
r2=0.39
P=0.003
r2=0.57
P=0.14
r2=0.77
P<0.0001
r2=0.29
P=0.13
r2=0.42
P=0.001
LAD-proximal
LAD-mid
non LAD-mid
LAD-distal
non LAD-proximal
non-LAD-distal
②Δ FFRCT
0.5
0.4
0.3
0.2
0.1
0
0
200
400
600
800
1000
ΔFFRCT
maxLCBI4mm
0.5
0.4
0.3
0.2
0.1
0
0
200
400
600
800
1000
ΔFFRCT
maxLCBI4mm
0.5
0.4
0.3
0.2
0.1
0
0
200
400
600
800
1000
ΔFFRCT
maxLCBI4mm
0.5
0.4
0.3
0.2
0.1
0
0
200
400
600
800
1000
ΔFFRCT
maxLCBI4mm
0.5
0.4
0.3
0.2
0.1
0
0
200
400
600
800
1000
ΔFFRCT
maxLCBI4mm
0.5
0.4
0.3
0.2
0.1
0
0
200
400
600
800
1000
ΔFFRCT
maxLCBI4mm
r2=0.69
P<0.0001
r2=0.24
P=0.40
r2=0.04
P=0.38
r2=0.24
P=0.006
r2=0.21
P=0.04
r2=0.004
P=0.87
